# Supplementary material for: Individual variability and versatility in an eco-evolutionary model of avian migration
Source: Proc Biol Sci. 2020 Nov 4;287(1938):20201339. doi: 10.1098/rspb.2020.1339 (PMC7735267; doi:10.1098/rspb.2020.1339)
Supplement: Supplementary Material [file rspb20201339supp1.pdf]

Proceedings of the Royal Society B: Biological Sciences doi: 10.1098/rspb.2020.1339

Individual variability and versatility in an eco-evolutionary model of avian migration

Kira E. Delmore†, Benjamin M. Van Doren†, Greg J. Conway, Teja Curk, Tania Garrido-Garduño, Ryan R. Germain, Timo Hasselmann, Dieter Hiemer, Henk P. van der Jeugd, Hannah Justen, Juan Sebastian Lugo Ramos, Ivan Maggini, Britta S. Meyer, Robbie J. Phillips, Magdalena Remisiewicz, Graham C. M. Roberts, Ben C. Sheldon, Wolfgang Vogl, Miriam Liedvogel

† These authors contributed equally to this work.

Supplementary Material

Additional analysis of light data

Package `FLightR` uses the slope of the light curve around twilight to estimate locations and is sensitive to data quality. In our dataset, several devices experienced shading due to mantle feathers covering the light sensor, especially after the summer molt. Geolocators with shorter "light pipes" ("-7" models, see Table S1) or with the sensor on the body of the device (deployed in Poland, see Table S1) were prone to this issue, whereas devices with a light sensor affixed to a 11-mm "light stalk" ("-11" models) rarely experienced shading. We performed an automated step to remove highly shaded light curves. For each twilight event, we took the mean of all "log.light" values returned by `FLightR` and removed twilights with values < 1. We removed no more than 10% of twilights with this method. This approach frequently improved performance, but we were unable to obtain `FLightR` tracks for 6 heavily shaded devices. These were excluded from the `FLightR` timing analysis.

Using the *siteEstimate* function in `GeoLight`, we obtained location estimates for all birds, including those for which `FLightR` had failed. For devices deployed in summer, we used twilights from 15 December to 15 January to estimate wintering locations. For devices deployed in winter, we used twilights from 1 June to 1 August to estimate summer breeding locations. In both cases, we set these time periods in mid-winter and mid-summer, when they are least likely to overlap with spring and autumn movements. We used the same time window for all birds to obtain comparable locations across individuals.

We set calibration periods by visually inspecting plots of the log of observed versus expected light slopes for the deployment site over time (*plot_slopes_by_location* function in `FLightR`). When a bird moves away from the deployment site, the observed and expected slopes visually diverge. For some individuals, visual resighting data were available after deployment and before recapture to aid calibration. After running `FLightR`, we refined calibration periods if necessary. Some devices had insufficient calibration time. In these cases, and cases where the resulting track showed clear signatures of poor calibration (e.g. latitudinal drift during stationary periods or widely varying location estimates), we used a global calibration made from the combined data of all devices. For this global calibration, we used a linear model to estimate the overall mean calibration slope, accounting for the magnitude of shading to the light sensor. We did not include devices that lacked light pipes or light stalks, which made the light data qualitatively different from those collected by the other devices.

In `GeoLight`, we used the same calibration periods as for `FlightR`, with one additional refining step: we used *siteEstimate* to estimate the location of deployment and compared the result to the actual deployment location; if a lower or higher sun angle ($\pm 0.25^{\circ}$ increments) resulted in a more accurate estimate of the deployment site, we used the adjusted sun angle.

We validated `FLightR` timing estimates using simple longitude coordinate output from `GeoLight` (*crds* function), which we used to derive alternative measures of migration timing across an east-west axis. With this method, we considered a bird to be halfway through its migration when its estimated longitude was closer

to the longitude of its destination than its origin. We defined the start of migration as the time when a bird crossed a threshold from its starting longitude and did not return. Our threshold was defined as 10% of the difference between origin longitude and destination longitude. We defined the end of migration as the point when a bird crossed to within 10% of its destination longitude. We expected migration timing estimated from longitude data to be most comparable to `FLightR` estimates for birds that primarily used east-west routes. For birds that primarily moved along a north-south axis, the component of movement across longitudes is small relative to the component across latitudes. Therefore, we excluded birds with strongly southerly migration directions (150º–210º) from this validation. The timing of spring migration was consistent across methods (all Spearman ρ>0.78). In autumn, ρ ranged from 0.62 to 0.78.

Permits

In Austria, fieldwork was approved by the institutional ethics and animal welfare committee and the national authority (GZ 68.205/0048-WF/V/3b/2016) according to §§ 26ff. of Animal Experiments Act, Tierversuchsgesetz 2012 – TVG 2012. Permit numbers: GZ BMWFW-68.205/0048-WF/V/3b/2016 and BMWFW-68.205/0139-WF/V/3b/2016 (AT), UID: ATU36801500, MA22-24411/2016 (Wien): BHBR-I-7100.00-69/2016-13 (VA), ABT13-53V-10/1998-42 (Steiermark), 205-05RI/549/58/7-2016 (Salzburg), N-2016-197947/8-Pin (Oberösterreich), VL3-NS-3068/2016 /005/2016) (Kärnten, Villach), SV19-ALL-938/2016 (004/2016) (Kärnten, St Veit), SP3-NS-2823/2016 (007/2016) (Kärnten, Spittal), HE3-NS-1280/2016 (005/2016) (Kärnten, Hermagor), FE3-NS-2127/2016 (006/2016) (Kärnten, Feldkirchen), 5/N.AB-10120-8-2016 (Burgenland), RU5-BE-286/011-2016 (Niederösterreich). In the UK, geolocator deployments were approved by the University of Oxford Animal Welfare Ethical Review Body. Work was conducted under licenses from the British Trust for Ornithology, approved by the Special Methods Technical Panel. In Poland, work was approved by the General Directorate for Environmental Protection within the permit to capture and ring wild birds (DZP-WG.6401.03.36.2015.km, DZP-WG.6401.03.98.2016.km, DZP-WG.6401.03.97.2017.jro, DZP-WG.6401.03.2.2018.jro). In Germany, the permit was issued by the Regierung von Mittelfranken, Bavaria. Permit number: 54-2532.1-13/14. In the Netherlands, the permit was issued by the Centrale Commissie Dierproeven. Permit number: AVD801002016519 valid 27-6-2016 through 31-5-2021.

Table S1: Geolocator deployment summary. All devices manufactured by Migrate Technology Ltd. Nylon material refers to 1 mm nylon braid for harnesses, viton refers to 0.6 mm viton rubber cord, and elastic refers to 0.7-0.8 mm stretch elastic. "Deploy", "Return", and "Recover" respectively refer to the number of devices deployed, the number of birds that were observed to have returned with devices, and the number of devices ultimately recovered from returning birds and successfully recaptured. "Controls Marked" and "Controls Returned" show the number of individuals that were marked with color-rings (without a geolocator) and were subsequently resighted.

Region	Year	Deploy	Return	Recover	Controls Marked	Controls Returned	Material	Device
Austria	2016	202	24 (5 viton)	24	0	0	nylon, viton	P65Z1top2end-11
Austria	2017	159	28	27	0	0	nylon	P50Z11-11
Austria	2018	15	4	3	0	0	nylon	P65Z1top1-11
Netherlands	2016	61	5	5	58	8	nylon	P50B1-11
Netherlands	2017	61	8	7	0	0	nylon	P50B1-11
Netherlands	2018	67	14	13	0	0	elastic	P30Z11-7-DIP-NOT; P65B1-7-NOT
Poland	2015	12	1	1	0	0	nylon	W30Z11-DIP-NOT; W65B1-DIP NOT
Poland	2016	9	1	1	0	0	nylon	W30Z11-DIP-NOT; W65B1-DIP NOT
Poland	2017	12	4	4	0	0	nylon	W65B1-DIP NOT; W30Z11-DIP-NOT
Poland	2018	20	4	3	0	0	nylon	W65B1-DIP NOT; W30Z11-DIP-NOT
S Germany	2018	57	7	5	0	0	elastic	P30Z11-7-DIP-NOT; P65B1-7-NOT
UK	2016-17	36	8	6	34	7	elastic	P50Z11-11-NOT
UK	2017-18	48	11	7	56	13	elastic	P50Z11-7-DIP-NOT
UK	2018-19	47	15	11	51	8	elastic	P50Z11-11-NOT

Table S2: Model results comparing migration timing in the migratory divide between SW and SE phenotypes and between intermediate (S) and SW/SE phenotypes. Log-transformed variables indicated by "(log)".

Contrast	Season (response)	Estimate	SE	df	t-ratio	P-value
SW vs. SE	Spring start	3.14	7.41	23	0.42	0.676
SW vs. SE	Spring middle	3.39	7.21	23	0.47	0.643
SW vs. SE	Spring end	3.48	5.05	22	0.69	0.498
SW vs. SE	Autumn start	9.23	6.42	27	1.44	0.162
SW vs. SE	Autumn middle	9.24	6.03	28	1.53	0.137
SW vs. SE	Autumn end	17.30	7.71	29	2.24	0.033
SW vs. SE	Autumn duration (log)	0.53	0.71	25	0.74	0.464
SW vs. SE	Spring duration (log)	-0.78	0.56	23	-1.41	0.172
SW vs. SE	Autumn speed (log)	-0.40	0.69	25	-0.59	0.562
SW vs. SE	Spring speed (log)	0.92	0.55	23	1.67	0.109
S vs. SW & SE	Spring start	-14.60	5.49	23	-2.66	0.014
S vs. SW & SE	Spring middle	-12.97	5.35	23	-2.42	0.024
S vs. SW & SE	Spring end	-8.88	3.48	22	-2.55	0.018
S vs. SW & SE	Autumn start	1.57	4.62	27	0.34	0.737
S vs. SW & SE	Autumn middle	-2.45	4.02	28	-0.61	0.548
S vs. SW & SE	Autumn end	-9.52	5.07	29	-1.88	0.071
S vs. SW & SE	Autumn duration (log)	-0.82	0.54	25	-1.53	0.139
S vs. SW & SE	Spring duration (log)	0.18	0.42	23	0.42	0.680
S vs. SW & SE	Autumn speed (log)	0.21	0.51	25	0.41	0.684
S vs. SW & SE	Spring speed (log)	-0.66	0.42	23	-1.56	0.133
Breeding latitude	Spring end	-5.34	2.85	22	-1.87	0.075
Breeding latitude	Autumn middle	-5.40	2.80	28	-1.93	0.064
Breeding latitude	Autumn end	-9.01	3.55	29	-2.54	0.017
Year (F-test)	Spring start	-	-	-	-	0.048
Year (F-test)	Spring middle	-	-	-	-	0.008
Year (F-test)	Spring end	-	-	-	-	0.012

Table S3: Model results comparing migration timing of British winterers (NW migrants) to SW migrants. All models tested for timing differences between NW and SW phenotypes; other predictor variables were removed if P>0.1 and are therefore omitted from the table. Log-transformed variables indicated by "(log)". NW and SW phenotypes differed more consistently in spring timing measures than in autumn. Likewise, protandry was evident only in spring, and breeding longitude was most strongly associated with the timing of spring migration. Breeding latitude did not show strong associations with any timing trait. Year effects were evident only in autumn, implying higher inter-annual consistency in spring.

Predictor	Season (response)	Estimate	SE	df	t-ratio	F-value	P-value
NW vs. SW	Spring start	-7.16	2.39	45	-3.00	-	0.004
NW vs. SW	Spring middle	-7.50	2.36	46	-3.17	-	0.003
NW vs. SW	Spring end	-9.83	2.46	46	-3.99	-	<0.001
NW vs. SW	Autumn start	-1.62	4.87	52	-0.33	-	0.741
NW vs. SW	Autumn middle	5.93	4.26	52	1.39	-	0.17
NW vs. SW	Autumn end	-11.80	6.40	51	-1.84	-	0.071
NW vs. SW	Autumn duration (log)	-0.58	0.36	51	-1.61	-	0.114
NW vs. SW	Spring duration (log)	-0.80	0.24	46	-3.28	-	0.002
NW vs. SW	Autumn speed (log)	0.33	0.45	50	0.72	-	0.476
NW vs. SW	Spring speed (log)	-0.08	0.22	46	-0.35	-	0.731
Male vs. Female	Spring start	-9.68	2.74	45	-3.53	-	0.001
Male vs. Female	Spring middle	-9.28	2.73	46	-3.40	-	0.001
Male vs. Female	Spring end	-9.80	2.84	46	-3.45	-	0.001
Male vs. Female	Autumn middle	8.32	4.77	52	1.74	-	0.087
Breeding longitude	Spring start	1.26	0.20	45	6.31	-	<0.001
Breeding longitude	Spring middle	1.23	0.20	46	6.21	-	<0.001
Breeding longitude	Spring end	1.21	0.21	46	5.84	-	<0.001
Breeding longitude	Autumn end	0.80	0.38	51	2.10	-	0.041
Breeding longitude	Autumn duration (log)	0.09	0.03	51	3.08	-	0.003
Breeding longitude	Autumn speed (log)	-0.07	0.03	50	-2.73	-	0.009
Breeding latitude	Autumn end	-1.68	0.88	51	-1.92	-	0.061
Breeding latitude	Autumn speed (log)	0.14	0.07	50	2.01	-	0.05
Year (F-test)	Autumn start	-	-	-	-	7.74	<0.001
Year (F-test)	Autumn middle	-	-	-	-	7.77	<0.001
Year (F-test)	Autumn end	-	-	-	-	2.57	0.064
Year (F-test)	Autumn duration (log)	-	-	-	-	2.30	0.089
Year (F-test)	Autumn speed (log)	-	-	-	-	3.54	0.021

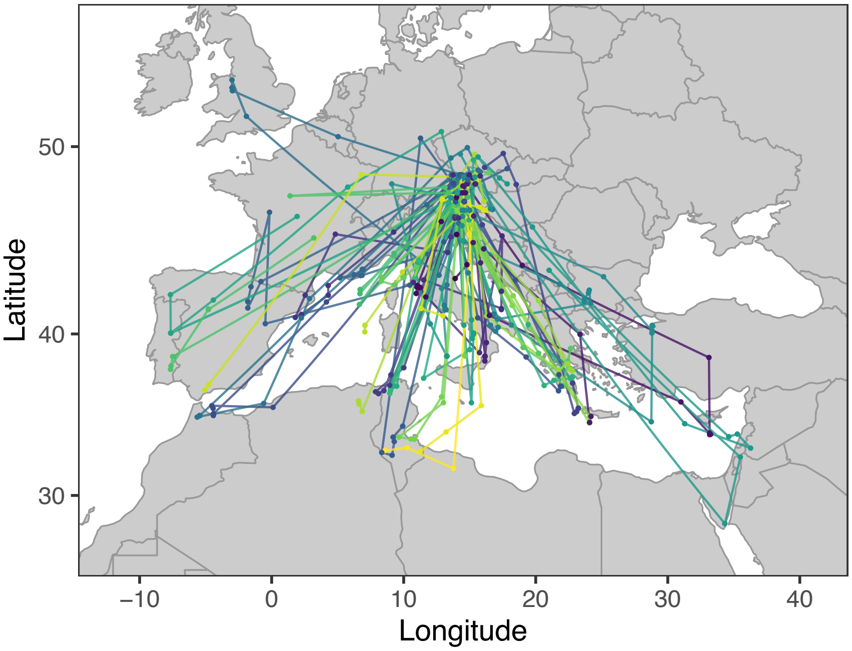

Figure S1: **Full tracks of blackcaps from the migratory divide**. Tracks estimated with `FLightR`, with each track in a different color. To reduce clutter, one point is shown for each month and error bars are omitted. `FLightR` estimated some wintering locations at slightly higher latitudes than the *siteEstimate* function in `GeoLight`; for example, some `FLightR` tracks that end in the southern Balkan Peninsula have `GeoLight` estimates on the northeast coast of Libya (Figure 1A). Note that headings over short distances are sensitive to the calibration used and may not be fully trustworthy.

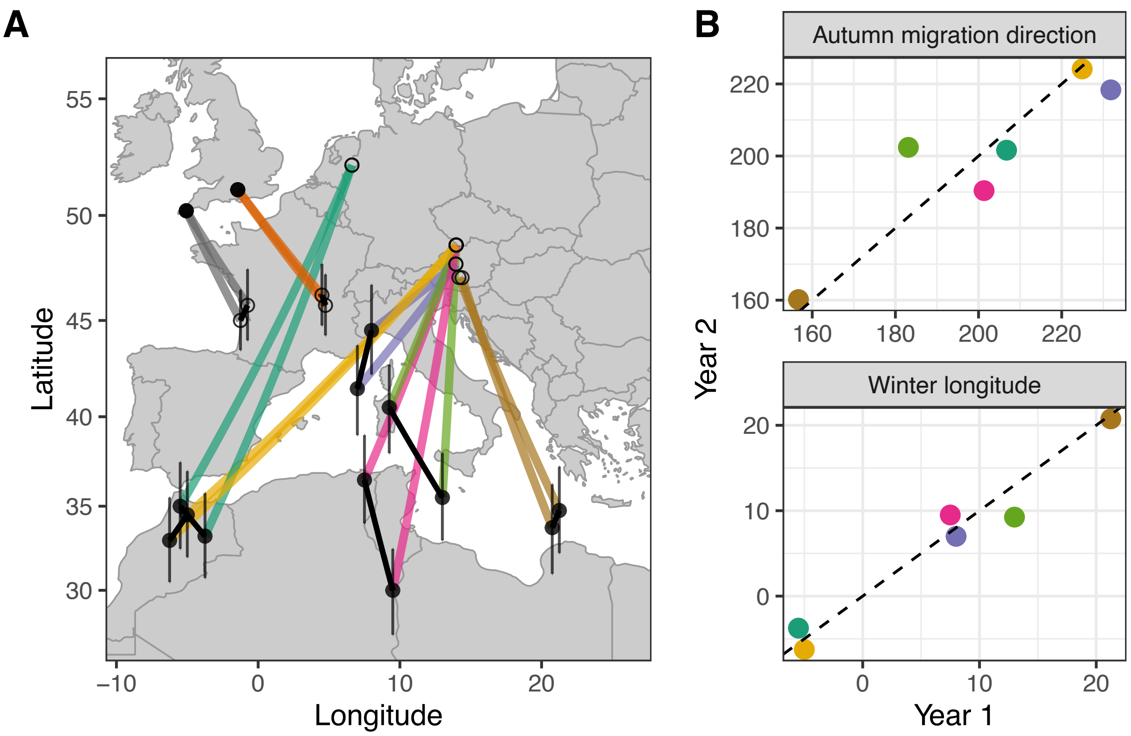

Figure S2: **Repeatability of migratory phenotypes within individuals.** **(A)** Each color represents one individual tracked over two subsequent years, with solid black lines connecting location estimates for the same individual. Breeding and non-breeding sites and error bars as in Figure 1. For the two British winterers, our repeated location estimates were very similar (59 and 92 km apart, respectively), strongly suggesting that they bred in the same area. **(B)** Migratory phenotype estimates for individuals tracked from continental Europe for two years (excluding those tagged in Britain). The dashed line is the identity line. We estimated repeatability in winter longitude as R [95% CI]=0.99 [0.96,1] and repeatability in migration direction as R [95% CI]=0.91 [0.77,1]. The winter location estimates for these individuals averaged 385±253 km apart in consecutive winters.

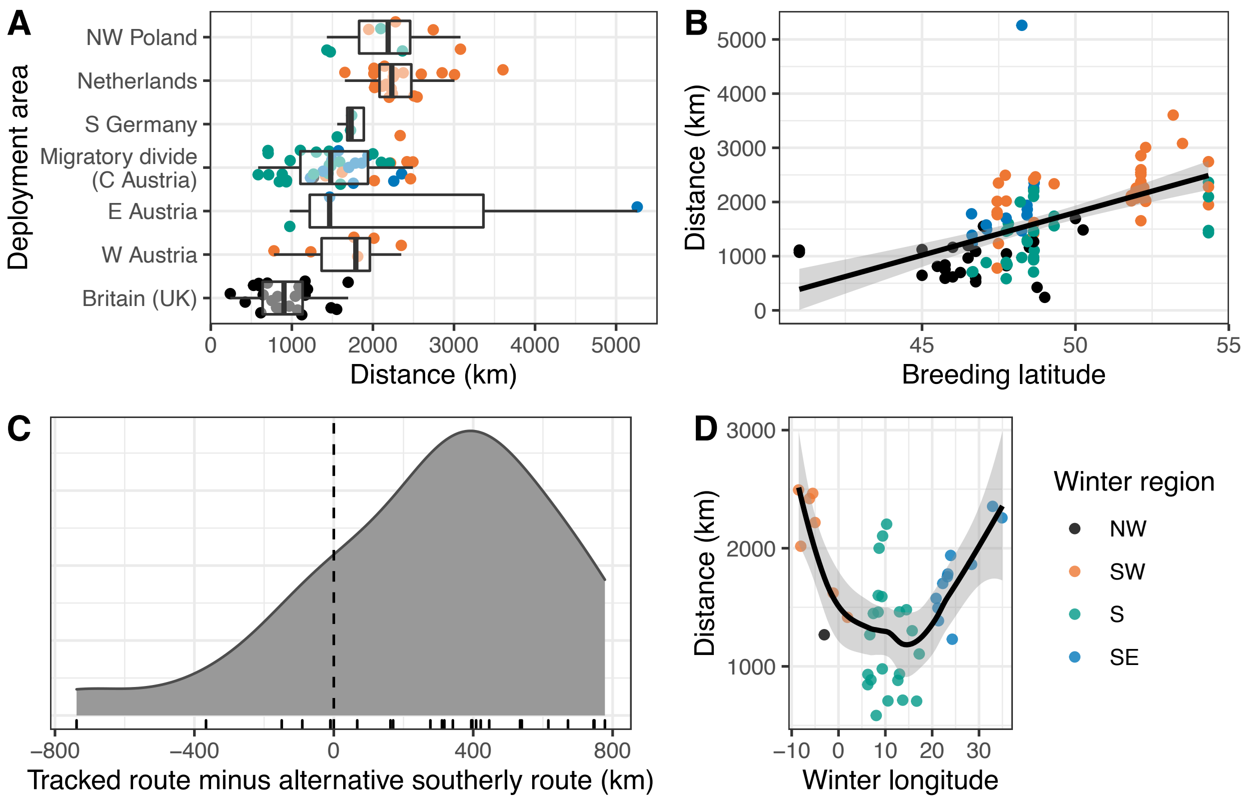

Figure S3: **Migration distances.** Colors indicate SW (orange)/intermediate (green)/SE (blue)/NW (black) autumn migratory phenotypes, categorized by wintering location. **(A)** Boxplots showing the distance between breeding and wintering sites for all blackcaps tracked, by deployment area. **(B)** Migration distance by breeding latitude, for all blackcaps tracked. **(C)** To determine how far a British overwintering blackcap would need to fly if it selected an alternative southerly route instead of a northerly route to the UK, we calculated the distance from the breeding site of each British winterer to the 10 closest wintering locations of tracked continental breeders. For 19 out of 25 cases, the tracked route to the UK was longer than the average of the 10 possible southerly routes, often by 400-600 km. Values shown are the difference between the observed migration distance and the average of the distances to the 10 closest tracked individuals that wintered in traditional southerly areas, instead of in the UK. **(D)** Migration distance by wintering longitude for blackcaps tracked within the migratory divide only. Individuals with intermediate directions had the shortest migration distances.
